# A flexible method to model HIV serodiscordance among couples in Mozambique

**DOI:** 10.1371/journal.pone.0172959

**Published:** 2017-03-02

**Authors:** Adelino J. C. Juga, Niel Hens, Nafissa Osman, Marc Aerts

**Affiliations:** 1 Department of Mathematics and Informatics, Faculty of Sciences, Eduardo Mondlane University, Maputo, Mozambique; 2 I-BioStat, Hasselt University, Diepenbeek, Belgium; 3 Centre for Health Economic Research and Modelling Infectious Diseases, Vaccine and Infectious Disease Institute (VAXINFECTIO), University of Antwerp, Antwerp, Belgium; 4 Epidemiology and Social Medicine (ESOC), Faculty of Medicine and Health Sciences, University of Antwerp, Antwerp, Belgium; 5 Department of Obstetrics and Gynaecology, Maputo Central Hospital, Maputo, Mozambique; 6 Faculty of Medicine, Eduardo Mondlane University, Maputo, Mozambique; Azienda Ospedaliera Universitaria di Perugia, ITALY

## Abstract

Whereas the number of people newly infected by HIV is continuing to decline globally, the epidemic continues to expand in many parts of the world. As the HIV/AIDS epidemic has matured in many countries, it is believed that the proportion of new infections occurring within couples has risen. Across countries, including Mozambique, a sizeable proportion of couples with HIV infection are discordant. A serodiscordant couple is a couple in which one partner has tested positive for HIV and the other has not. To describe the HIV serodiscordance among couples, a variety of association measures can be used. In this paper, we propose the serodiscordance measure (SDM) as a new alternative measure. Focus is on the specification of flexible marginal and random effects models for multivariate correlated binary data together with a full-likelihood estimation method, to adequately and directly describe the measure of interest. Fitting joint models allows examining the effects of different risk factors and other covariates on the probability to be HIV positive for each member within a couple, and estimating common effects for both probabilities more efficiently, while accounting for the association between their infection status. Moreover, the interpretation of the proposed association parameter SDM is more direct and relevant and effects of covariates can be studied as well. Results show that the HIV prevalence for the province where a couple was located as well as the union number for the woman within a couple are factors associated with HIV serodiscordance. These findings are important for the Mozambican public health policy makers to design national prevention plans, which include policies to stimulate regular HIV testing for couples as well as adolescents and young adults, prior to getting married or living together as a couple.

## Introduction

In recent years, there has been increasing interest in the spread of HIV within stable sexual partnerships [1], since the HIV epidemic has matured in many countries and it is believed that the proportion of new infections occurring within couples has risen [2]. Evidence has shown that across countries, a sizeable proportion of couples with any HIV infection are discordant [2]. A serodiscordant couple is a couple in which one partner has tested positive for HIV and the other has not.

Evidence suggests that women have the greatest risk of contracting HIV within a marital relationship. Fewer attempts have been made to understand a man’s risk. But regions with generalised epidemics, high rates of serodiscordance among heterosexual couples in which the woman is HIV positive suggest that a man’s risk of marital HIV acquisition could also be substantial [3].

In Mozambique, in among 1.6 million people living with HIV/AIDS in 2009, around one in every ten couples were discordant. In 5% of all couples, both members were HIV positive (concordant positive), while in 10% of all couples, one member was HIV positive whilst the other was HIV negative (female or male discordant). The estimates of 2011 showed that the proportion of male discordant couples was similar to the proportion of female discordant couples [1]. By female discordant we refer to a couple where the woman was HIV positive while the man was HIV negative. A couple where the man was HIV positive whilst the woman was HIV negative is referred to as a male discordant couple.

Despite the empirical evidence pointing to their programmatic importance, serodiscordant couples are often overlooked or, at best, only vaguely addressed in many national prevention plans. This omission may stem not only from sensitivity surrounding HIV within couples but also from misperceptions about the extent of serodiscordance and failure to understand that it is possible to prevent transmission within a stable union once one partner has become infected [4].

Statistical methods and models to investigate risk factors associated with HIV serodiscordance among couples can be very helpful. Simple methods are based on the use of descriptive statistics as well as bivariate associations. Extensions of such descriptive analyses have been covered by (standard) logistic regression models. Kaiser *et al*. [5] developed two different models to assess factors associated with couple status, one for HIV discordance as the outcome and a second one with HIV concordance as the binary outcome. In the first model, concordant HIV-infected couples were excluded from the denominator and in the second model concordant uninfected couples were excluded from the denominator. Based on the 2007 Kenya AIDS Indicator Survey, they found that the following factors were independently associated with HIV-discordance: young age in women, increasing number of lifetime sexual partners in women, HSV-2 (herpes simplex virus type 2) infection in either or both partners, and lack of male circumcision. Independent factors for HIV-concordance included HSV-2 infection in both partners and lack of male circumcision. Fishel *et al*. [1] based their findings on an analysis including two logistic regression models. The first model compared concordant positive couples with all discordant couples, regardless of whether the discordant couple is male discordant or female discordant. The second multinomial logistic regression compares concordant positive couples separately to male discordant and female discordant couples. They concluded that HIV discordance of couples in Mozambique does not appear to have a strong association with whether or not the couple is polygynous, the amount of time passed since the couple last had sexual intercourse with each other, or risk factors for non-sexual transmission of HIV. Although there are some differences in the HIV status of couples by characteristics of interest, the differences do not outline a profile that makes discordant couples easy to distinguish from the general population.

Our approach is different in two ways. Instead of using two separate logistic models, we propose to use genuine bivariate models for correlated binary data, allowing one to fit separate logistic regression models in one single analysis while taking into account the correlation between both binary responses (HIV status of man and woman within a couple). Moreover we propose to reparametrize the model such that serodiscordance itself is a model parameter, allowing to investigate the factors affecting the serodiscordance in a direct way. The particular measure of serodiscordance proposed in this paper is inspired by the conditional synchrony measure (CSM) as introduced by [6] to measure synchrony in neuronal firing.

There exist different families of models for correlated binary data: so-called marginal, population averaged models and conditional models. Full likelihood models such as the bivariate Dale model [7] and alternative models estimated by the method of moments (Generalized Estimating Equations, GEE, [8]) are of the first type. Generalized linear mixed models [9], such as the logistic-normal model, are based on the use of random effects and constitute a second type of models. Which model family or combination of types is to be preferred depends on the design of the study and on the research question(s) of interest.

In this paper, we will propose the combination of a reparametrized version of the bivariate Dale model together with multivariate random effects with different covariance structures to take into account the design of the study. Focus is on the specification and estimation of appropriate covariate models for all model components including the serodiscordance parameter. The models were designed to investigate in particular the relationship/association between the HIV status of woman and man within a couple and the risk factors associated with HIV serodiscordance among couples.

The paper is organized as follows. A first section on materials and methods provides information on the INSIDA survey with some details about the survey design and the variables used in this study. The statistical models are introduced together with a new measure of serodiscordance. The use of survey weights and the model building strategy is briefly discussed. The second section summarizes the results of the application of the proposed methodology on the INSIDA data and the paper ends with conclusions and a final discussion.

## Materials and methods

### Survey design

We used data from the 2009 National Survey of Prevalence, Risk Behavioural and Information about HIV and AIDS (INSIDA, [10]). This survey is a cross-sectional two-stage survey, carried out by the National Institute of Health in collaboration with the National Bureau of Statistics of Mozambique. It was the first survey designed to collect comprehensive data on the prevalence of HIV infection, knowledge, attitude, behaviour risk factors and access to information on HIV and AIDS in the Mozambican population. Of particular interest in this survey was the HIV status of cohabiting couples. Access to the data can be requested by logging in at http://dhsprogram.com/data/dataset/Mozambique_Standard-AIS_2009.cfm?flag=0 and by submitting a research project proposal.

Stratification and cluster sampling methods were applied to ensure that for each province inference was possible with nearly the same precision. Moreover, two-stage sampling was also used to access individuals within households. Enumeration Areas (EAs), households and individuals were Primary Sampling Units (PSU), Secondary Sampling Units (SSU) and Tertiary Sampling Units (TSU) respectively. Under stratification, 11 provinces were considered as stratums. Moreover, 270 EAs were selected in all provinces from a total of 45000 EAs defined according to the cartography of general census, 2007. Out of selected 270 EAs, 122 were urban and 148 rural. Then a fixed number of households were systematically selected within each EA in the second stage. In this phase, 22 households from urban EAs and 24 from rural EAs were selected [1]. Men and women aged between 15–64 years were eligible to participate in an individual interview and to provide a blood sample for the HIV test. For ethical reasons, it was not asked whether the respondents knew their HIV status [1]. To identify couples, for each woman that was married or lived together with her male partner, an attempt was made to match her with her husband/partner using his household line number. A confirmation was then made by checking the man’s interview information that was named by the woman. If a man was polygamous (married or living with more than one wife/woman) he may appear in the database multiple times, once for each wife [1]. 334 men appeared to be polygamous. Out of 6190 households, 10(0.16%) of them had two couples and only 1 household had 3 couples.

### Description of variables

Table 1 shows a brief description of all variables used in the final model. None of them has missing values. The HIV status (infected yes/no) for each member within a couple constitute the (binary) response variables of interest. The following covariates appear in the final model with fixed effects. The variables union number for man/woman refer to whether the respondent has been married or lived with a woman/man once or more than once. Wealth index refers to the economic status of the couple while condom used by man refers to whether the male respondent of the couple used a condom the last time he had sexual intercourse with the other partner. The HIV prevalence for province was categorized into three categories using cutpoints of 5 and 15% [1]. Finally, heterogeneity across the randomly selected EAs was modelled using random effects.

**Table 1:** INSIDA survey: basic description of variables used in the final model.

Variables	Type	Levels
**At the individual level**		
HIV status for woman	Binary	0: HIV Negative (refence category)
		1: HIV Positive
HIV status for man	Binary	0: HIV Negative (reference category)
		1: HIV Positive
Union number for woman	Binary	1: once (reference category)
		2: more than once
Union number for man	Binary	1: once (reference category)
		2: more than once
**At the level of the couple**		
Condom used by man	Binary	1: Used(reference category)
		2: Not used
Wealth index	Categorical	1: Poorer
		2: Middle
		3: Richer (reference category)
**At the level of the province**		
HIV prevalence of province	Categorical	1: < 5%(reference category)
		2: 5%–15%
		3: > 15%

### Joint models for bivariate binary outcomes

In this section the statistical model is introduced and different measures of association are discussed. A new measure of association, the serodiscordance measure, is introduced and embedded in the statistical model. Next, the model is extended with random effects accounting for heterogeneity across enumeration areas. Finally more information is provided about the use of sample weights accounting for the survey design. Finally the model building strategy is briefly described.

#### Joint marginal model

Let *y* = (*y*_1_, *y*_2_) denote the HIV status of a couple, woman and man respectively, and *x* a vector of covariates (including the constant 1 associated with the intercept). Some of the covariates are couple-specific (such as wealth index), whereas others are individual specific (union number). But any variable is considered to have a potential effect on the joint distribution of *y* = (*y*_1_, *y*_2_). So, e.g. condom use by man could have an effect on the probability that the HIV status of the man is positive, on that of the woman as well and possibly on the association between both statuses (while correcting for all other covariates in the model).

The model arises from the decomposition of the joint probabilities (given a covariate pattern *x*)

$$\pi_{j_{1}j_{2}}=P\left(y_{1}=j_{1},y_{2}=j_{2}|x\right),\;\;j_{1},j_{2}=0,1,$$ (1)

into the marginal probabilities of each couple member’s status to be positive, *π*_*F*_ = *P*(*y*_1_ = 1) = *π*_10_ + *π*_11_ and *π*_*M*_ = *P*(*y*_2_ = 1) = *π*_01_ + *π*_11_, together with an association parameter *ϕ*. The effect of covariates on *π*_*F*_, *π*_*M*_ and *ϕ* can be modelled as follows (extending the logistic regression model):

$$\begin{array}{c} h_{1}\left(\pi_{F}\right)=\beta_{1}^{T}x,\\ h_{2}\left(\pi_{M}\right)=\beta_{2}^{T}x,\\ h_{3}\left(\phi \right)=\beta_{3}^{T}x, \end{array}$$ (2)

where *h*_1_, *h*_2_ and *h*_3_ are appropriate link functions. Typical choices for *h*_1_ and *h*_2_ are the logit, probit, cloglog link (see e.g. [11]). Here we focus on the logit link as it allows an appealing interpretation of the effects of the covariates in terms of odds ratios. The choice of the link function *h*_3_ is related to the particular choice of the association parameter: the correlation coefficient $\rho =\left(\pi_{11}-\pi_{F}\pi_{M}\right)/\sqrt{\pi_{F}\left(1-\pi_{F}\right)\pi_{M}\left(1-\pi_{M}\right)}$, the odds ratio OR = (*π*_00_*π*_11_)/(*π*_10_*π*_01_) or just *π*_11_, or any other choice that more clearly represents the parameter of interest, being the serodiscordance in our setting.

The model components Eq (2) can be embedded in different frameworks of estimation and inference. The standard application of Generalized Estimating Equations (GEE, moment estimation, see e.g. [11]) focuses on the two model components for *π*_*F*_ and *π*_*M*_ while treating the “intra-couple” correlation as a nuisance parameter, not allowing to incorporate any model $h_{3}\left(\phi \right)=\beta_{3}^{T}x$. Extensions such as Alternating Logistic Regression(ALR) proposed by [12] extend standard GEE with the possibility to include a model $h_{3}\left(\phi \right)=\beta_{3}^{T}x$ and uses the OR rather than the correlation coefficient. But existing software is limiting the type of such models one can fit. Here we opt however for full Maximum Likelihood (ML), as this paradigm allows modelling in principle any association parameter by any kind of covariate model and is able to combine fixed effects for the covariates with random effects to represent other sources of hierarchical heterogeneity in a conceptually straightforward way (such as the EAs in our application).

The joint probabilities *π*_*j*_1_*j*_2__ used to construct the multinomial (log-)likelihood are reparametrized in terms of *π*_*F*_, *π*_*M*_ and *π*_11_ as follows

$$\pi_{j_{1}j_{2}}=\left\{\begin{array}{ccccc} \pi_{11} & \text{if} & j_{1}=1 & \text{and} & j_{2}=1,\\ \pi_{F}-\pi_{11} & \text{if} & j_{1}=1 & \text{and} & j_{2}=0,\\ \pi_{M}-\pi_{11} & \text{if} & j_{1}=0 & \text{and} & j_{2}=1,\\ 1-\pi_{F}-\pi_{M}+\pi_{11} & \text{if} & j_{1}=0 & \text{and} & j_{2}=0. \end{array}\right.$$ (3)

The joint probability *π*_11_ that both partners of the same couple are HIV positive can be replaced, as the association parameter, by the correlation coefficient *ρ*, or by the odds ratio, the latter being the most natural choice for binary data. The multinomial likelihood with probabilities *π*_*j*_1_*j*_2__, via identities Eqs (2) and (3) written in terms of the regression parameters (*β*_1_, *β*_2_, *β*_3_), is the basis for ML estimation and inference.

Within this ML framework, model (2) with logit links for *π*_*F*_ and *π*_*M*_, and the log link for *ϕ* = OR corresponds to the Bivariate Dale Model (BDM) [7]. The reparameterisation formulas for *π*_11_ and OR are given by

$$\text{OR}=\frac{\pi_{11}\left(1-\pi_{F}-\pi_{M}+\pi_{11}\right)}{\left(\pi_{F}-\pi_{11}\right)\left(\pi_{M}-\pi_{11}\right)}$$ (4)

and, if OR ≠ 1

$$\begin{array}{c} \pi_{11}=\frac{1+\left(\pi_{F}+\pi_{M}\right)\left(\text{OR}-1\right)-{\left\{\left[1+\left(\pi_{F}+\pi_{M}\right){\left(\text{OR}-1\right)}^{2}\right]+4\text{OR}\left(1-\text{OR}\right)\pi_{F}\pi_{M}\right\}}^{\frac{1}{2}}}{\left[2(\text{OR}-1)\right]} \end{array}$$ (5)

and when OR = 1, *π*_11_ = *π*_*F*_*π*_*M*_. In general the OR is preferred for its interpretation and its orthogonality to marginal probabilities *π*_*F*_ and *π*_*M*_ (in the sense that the corresponding elements in the expected covariance matrix are zero). Although the OR is an attractive measure describing the association between binary variables, it treats concordant positive and concordant negative couples fully equally, inflating the OR as a measure for (con/dis)cordance. Furthermore, the magnitude of association between the two outcome may be misunderstood, leading to unrealistic estimates [13]. Indeed, as the majority of couples is concordant negative, the OR might be high even if there is only a small number of concordant positive couples. For example, for the INSIDA data, the overall sample estimates are ${\hat{\pi }}_{00}=0.84$ (95% CI [0.82,0.85]) and $\hat{\text{OR}}=14.75$ (95% CI [10.16,19.34]), so both very high, whereas the estimate for concordant positive couples is small: ${\hat{\pi }}_{11}=0.05$ (95% CI [0.04,0.06]). For the purpose of our particular research question, interest goes to couples being discordant (or concordant), given that at least one of both is positive. This type of association measure is introduced and discussed in the next subsection.

#### A new serodiscordance measure

In a completely different context of measuring synchrony in neuronal firing, [6] proposed a new measure of synchrony, the conditional synchrony measure CSM, which is the probability of two neurons firing together, given that at least one of the two is active. Faes *et al*. [6] state that, although the odds ratio is an attractive association measure with nice mathematical properties (such as the absence of range restrictions, regardless of the marginal probabilities), it is less suitable to quantify synchrony due to its symmetry, treating 0–0 matches of equal importance as 1–1 matches. The CSM is defined as

$$\text{CSM}=\frac{\pi_{11}}{\pi_{F}+\pi_{M}-\pi_{11}},$$

and translated to our application, it is the conditional probability that both, man and woman, are HIV positive, given that at least one of them, man or woman, is HIV positive.

As we are rather interested in discordance, we define the HIV serodiscordance measure SDM as the conditional probability that the couple is HIV discordant, given that at least one of them, man or woman, is HIV positive, or

$$\text{SDM}=1-\text{CSM}=\frac{\pi_{F}+\pi_{M}-2\pi_{11}}{\pi_{F}+\pi_{M}-\pi_{11}}.$$ (6)

Being a (conditional) probability, SDM takes values between 0 and 1, with higher values indicating more HIV discordance within the couple. The global sample estimate for the INSIDA data, $\hat{\text{SDM}}=0.6747$ (95% CI [0.63,0.72]), indicates a quite high amount of discordance. It is the objective to examine how the SDM depends on different factors, while accounting for the effects of (possibly) partly common, partly different factors on the marginal probability *π*_*F*_ and *π*_*M*_. A logit link function is used for *h*_3_ in the definition of the model components Eq (2), allowing to interpret effects of factors as odds ratios contrasting the probabilities to be discordant to that of being concordant, given that at least one member of the couple is HIV positive. The joint probability *π*_11_, used in the loglikelihood expressions based on Eq (3), can be expressed in terms of the SDM, *π*_*F*_ and *π*_*M*_ as follows

$$\begin{array}{c} \pi_{11}=\frac{1-\text{SDM}}{2-\text{SDM}}\left[\pi_{F}+\pi_{M}\right]. \end{array}$$ (7)

In the next section the fixed effects model (2) using association parameter *ϕ* = SDM and logit links for all three parameter models, is extended with random EA effects to accommodate heterogeneity across EAs.

#### Extension with random effects to capture heterogeneity at the EA level

Let *y*_*ij*_ = (*y*_*ij*1_, *y*_*ij*2_) denote the HIV status of a couple *j* = 1, …, *n*_*i*_ in enumeration area *i* with *n*_*i*_ sampled couples, *i* = 1, …, *N*. For the INSIDA data, the total number of enumeration areas is *N* = 270 and $\sum_{i=1}^{N}n_{i}=2159$ is the total number of couples. As *π*_*F*_, *π*_*M*_, and SDM are expected to be heterogeneous across EAs, model (2) has to be extended with an EA effect. Model (2) is therefore extended with EA random effects as follows, adding subscripts *i* and *j* and expressing that the covariates *x*_1,*ij*_, *x*_2,*ij*_ and *x*_3,*ij*_ can be possibly different subvectors of the full covariate/factor vector *x*_*ij*_,

$$\begin{array}{c} \text{logit}\left(\pi_{F,ij}\right)=\beta_{1}^{T}x_{1,ij}+b_{F,i},\\ \text{logit}\left(\pi_{M,ij}\right)=\beta_{2}^{T}x_{2,ij}+b_{M,i},\\ \text{logit}\left({\text{SDM}}_{ij}\right)=\beta_{3}^{T}x_{3,ij}+b_{D,i}, \end{array}$$ (8)

where

$$\left(b_{F,i},b_{M,i},b_{D,i}\right)∼N_{3}\left(0,\Sigma \right),$$ (9)

are distributed as a trivariate normal distribtion with mean zero-vector and covariance matrix Σ. Note that *x*_ℓ,*ij*_ (ℓ = 1, 2, 3) are vectors of covariates, some of these covariates are specific for the male/female individual, some specific for the couple, and some are at the level of province.

The variance components $\sigma_{F}^{2},\sigma_{M}^{2}$ and $\sigma_{D}^{2}$ quantify the degree of heterogeneity across the AEs for each of the three parameters. Different choices considered for the covariance matrix Σ = *VRV* with

$$V=\left(\begin{array}{ccc} \sigma_{F} & 0 & 0\\ 0 & \sigma_{M} & 0\\ 0 & 0 & \sigma_{D} \end{array}\right),$$

are based on different choices for the correlation matrix *R*.

(Partial) Correlated Random Effects

A first option is a fully correlated random effects structure with

$$R_{\text{COR}}=\left(\begin{array}{ccc} 1 & \rho_{FM} & \rho_{FD}\\ \rho_{FM} & 1 & \rho_{MD}\\ \rho_{FD} & \rho_{MD} & 1 \end{array}\right).$$

A reduced partial-correlated version assumes only one correlation component to be non-zero: *ρ*_*FM*_ ≠ 0 and *ρ*_*FD*_ = *ρ*_*MD*_ = 0, so HIV status and SDM random effects are not correlated.

(Partial) Shared Random Effects

Another simplified structure corresponds to *ρ*_*FD*_ = *ρ*_*MD*_ = *ρ*_*FM*_ = 1 in *R*_COR_ leading to *R*_COR_ = *J*_3_, the all-ones matrix. This implies a full-shared random effect, with *b*_*M*,*i*_ = (*σ*_*M*_/*σ*_*F*_)*b*_*F*,*i*_ and *b*_*D*,*i*_ = (*σ*_*D*_/*σ*_*F*_)*b*_*F*,*i*_. This shared version is simplified further by putting *σ*_*D*_ = 0, leaving only (two) shared random effects in the models for *π*_*F*,*ij*_ and *π*_*M*,*ij*_ and assuming SDM_*ij*_ to be constant across AEs (after correcting for covariates). This model will be referred to as the partial-shared random effects model.

Partial Equal Random Effects

A final reduction concerns setting *σ* = *σ*_*M*_ = *σ*_*F*_ in the partial-shared model, such that *b*_*M*,*i*_ = *b*_*F*,*i*_, resulting in the partial-equal random effects model.

Independent Random Effects

As a final model, consider *ρ*_*FD*_ = *ρ*_*MD*_ = *ρ*_*FM*_ = 0 resulting in *R*_COR_ = *I*_3_, the identity matrix, and corresponding to independent random effects.

#### Accounting for survey design using weights

Most of the sample designs for household surveys such as INSIDA are complex and involve stratification, multistage sampling, and unequal sampling rates. Such survey designs are often more appropriate as coverage of the entire population of interest is better achieved and are often practically more efficient for interviewing subjects [14]. But it is necessary to account for the particular survey design in the statistical analyses using appropriate weights. For details on the calculation of the weights as used in our analyses, we refer to [1].

#### Model building

For the selection of the covariates (shown in Table 1), the following model building strategy was followed. Ideally one would select the covariates in all three model components Eq (8) starting from the most complicated random effects type of model (9), but that appeared to be computationally not feasible. Therefore, as a pragmatic but reasonable alternative, in our view, stepwise regression was first applied to the two univariate logistic regression models for *π*_*F*_ and *π*_*M*_ separately. Later, these covariates were used in the joint marginal as well as in its extension to the AE-specific random effects models, hereby extending the logistic model for SDM in a stepwise forward manner. In a last step it was investigated whether the intercepts and some or all of the slopes in the two univariate logistic regression models for *π*_*F*_ and *π*_*M*_ could be taken in common. The final joint model was selected using the Akaike Information Criterion (smaller values of AIC indicate a better fit, [15]).

Data analysis was performed in SAS version 9.3 using PROC NLMIXED (code in Appendix 1).

## Results

As the selected set of covariates for the two univariate logistic regression models for *π*_*F*_ and *π*_*M*_ only differs in one covariate for each model component, it was decided to keep the set equal for both, allowing a more complete comparison and interpretation of the estimated effects on the probability to be HIV positive for the female and the male partner within the same couple. The covariates involved are: the HIV prevalence of the province, the union number of the woman and that of the man, the condom use by the man, and the wealth index. The stepwise procedure to identify the factors influencing the serodiscordance measure SDM led to significant effects of the HIV prevalence of the province, and the union number of the woman.

The model components Eq (8) with these selected sets of covariates were then fitted simultaneously with different (multivariate) random effects structures, capturing the heterogeneity across the EAs. Table 2 shows the values of -2×log-likelihood, the number of parameters, the AIC and BIC goodness of fit measures of all fitted models: the joint marginal model without random AE effects and a subset of all random effects models, with different and with partly common slopes for the models for *π*_*F*_ and *π*_*M*_. The last two columns shows the models’ ranks according to AIC and BIC. The independent random effects and the full or partial random effects models did not convergence. So the results and the discussion is limited to the full-, partial-shared and partial equal random effects models.

**Table 2:** Comparison of marginal model, and full-shared, partial-shared and partial-equal random effects models, all without or with common intercept and common slope for HIV prevalence and wealth index for the models for *π*_*F*_ and *π*_*M*_. The column ‘-2ll’ shows the values of -2×log-likelihood; the column ‘#Par’ shows the number of parameters and the columns ‘Rank’ refers to the ranking of the models according to the AIC and BIC criterion.

Model	-2ll	# Par	AIC	BIC	Rank_*A*_	Rank_*B*_
MM (marginal model)	2584.8	20	2624.8	2738.3	8	8
CE-MM (common effects MM)	2590.4	15	2620.4	2705.5	7	7
FS (full-shared RE model)	2534.2	23	2580.2	2662.8	6	6
CE-FS (common effects FS)	2538.0	18	2574.0	2638.7	4	3
PS (partial-shared RE model)	2534.2	22	2578.2	2657.2	5	5
CE-PS (common effects PS)	2538.1	17	2572.1	2633.2	2	2
PE (partial-equal RE model)	2534.7	21	2576.7	2652.1	3	4
CE-PE (common effects PE)	2539.8	16	2571.8	2629.3	1	1

According to AIC and BIC the best model is the partial-equal random effects model with common effects (CE-PE) for the HIV prevalence of the province and the wealth index, rather closely followed by the partial-shared random effects model with the same common effects (CE-PS). Based on the likelihood ratio test (LRT), the null hypothesis of equal random effects *σ*_*M*_ = *σ*_*F*_ cannot be rejected at level 0.05 (p-val = 0.19). So, the CE-PE model is taken as final model.

The estimates of all 16 parameters of the CE-PE model and their standard error estimates are shown in Table 3. In the following discussion 95% confidence intervals for the odds ratio (OR-CI) are also shown. First of all, the probability to be HIV positive increases with the HIV prevalence in the province, for both women and men in the same way, as to be expected. As compared to a province with less than 5% prevalence, the odds to be HIV positive increases with a factor of about 6.9 when living in a province with more than 15% prevalence (OR-CI [4.10,11.62]). The effect of wealth index is also identical for women and men: as compared to the “richer” reference category, the odds to be HIV positive is estimated to be about 32% lower for the “middle” category, and 46% lower for “poorer” category (OR-CI [0.48,0.96] and [0.38.0.75] respectively). In Mozambique it is quite usual that one (or both but especially the male) partner within a richer couple has multiple extramarital partners or even visits sex workers, increasing the risk for HIV infection [16], [17]. The effects of union number and condom use are different for women and men. Whereas condom use has no significant effect on the odds for men to be HIV positive, not using a condom is estimated to increase the odds for women to be HIV positive with about 90% (OR-CI [1.16,3.12]). That the male partner has been married or lived with a female partner more than once has no significant effect on the odds for the woman to be HIV positive, but it increases the odds for the man to be HIV positive with about 45% (OR-CI [1.09,1.93]). A female partner being married or having lived with a male partner more than once has a much higher odds to be positive (increase of about 121%, OR-CI [1.61,3.02]) and the same holds for her male partner, though with a more limited increase of about 56% (OR-CI [1.13,2.16]). For most combinations of the covariate values the estimated probability to be HIV positive is higher for the female partner, in line with what is known. For the female partner the fitted probability to be HIV positive varies between 1.55% and 48.50% depending on the covariate pattern; for the male partner between 1.55% and 31.86% which is less but still substantial. The above discussed OR estimates and confidence intervals are for a given EA, and the estimated probabilities for a “central” EA with specific EA-effect equal to 0.

**Table 3:** Parameters estimates and standard error estimates for the CE-PE model.

Effect	HIV Woman	HIV Man	SDM
	Estimates(SE)	Estimates(SE)	Estimates(SE)
**Intercept**	-3.53(0.254)*		1.67(0.440)*
**HIV prevalence**			
5–15%	1.10(0.248)*		-0.56(0.452)
> 15%	1.93(0.264)*		-1.08(0.455)*
**Union number woman**			
More than once	0.79(0.189)*	0.44(0.165)*	-0.57(0.232)*
**Union number man**			
More than once	0.11(0.150)	0.37(0.144)*	-
**Condom use man**			
Not used	0.64(0.251)*	0.03(0.248)	-
**Wealth index**			
Poorer	-0.62(0.172)*		-
Middle	-0.39(0.177)*		-
**Variance component**			
*σ*^2^	0.46(0.115)†		

* Significant at 5% level (Wald test)

^†^ Significant at 5% level, using $\chi_{0,1}^{2}$ mixture

The null hypothesis *H*_0_: *σ* = 0 is rejected at 5% level, using a $\chi_{0,1}^{2}$ mixture, indicating a significant EA effect on the probability to be HIV positive (common to both members of the couple in that EA) and implying highly varying probabilities to be HIV positive across EAs. For an EA with an extreme negative EA-effect $-2\hat{\sigma }=-1.356$ the estimated probability to be HIV positive for the female partner varies between 0.4% and 19.50% (depending on the covariate pattern); for the male partner between 0.4% and 10.75%. On the other hand, for an EA with an extreme positive EA-effect $2\hat{\sigma }=1.356$ the estimated probability to be HIV positive for the female partner varies between 5.77% and 78.52% (depending on the covariate pattern); for the male partner between 5.77% and 64.48%.

The odds for a couple to be serodiscordant decreases with the HIV prevalence of the province. As compared to a province with less than 5% prevalence, the odds for a couple to be serodiscordant decreases with 66% when living in a province with more than 15% prevalence (OR-CI [0.14,0.84]). In other words, given that at least one of the two partners is positive, the probability that both are positive increases with the HIV prevalence of the province where the couple is living. The odds of serodiscordance also decreases when the woman has been married or living with a man more than once, with an estimated decrease of about 43% (OR-CI [0.36,0.89]). Given that one of the two partners is positive, the probability that the other is not varies between 50.50% and 84.16%. Note that, according to the CE-PE model, the probability to be serodiscordant does not vary across EAs, in contrast to the probabilities for men and women to be HIV positive.

## Discussion and conclusions

In this paper, a new serodiscordance measure SDM is defined as the conditional probability that both partners differ in their HIV status, given that one of them is positive. Together with the probabilities to be HIV positive for man and woman, the SDM parameter is embedded in a bivariate statistical model with fixed and random effects for covariates and design variables on each of the three parameters. A (final) model with significant fixed effects for the HIV prevalence of the province, the union number of woman and man, condom use and wealth index, and a significant random effect for the EAs was fitted using data from the 2009 INSIDA survey. An extension of the current analysis with more recent survey data and extending the model to investigate any time trend is a very interesting and relevant topic for further research.

In Zambia, a retrospectively study of 65 couples to estimate the likely origin of HIV infection, found that at least one quarter of cases of HIV infection in recently married men were acquired from extramarital partnerships, and for both men and women, less than one half of cases of HIV infection were acquired from their spouse/husband [4]. In addition, they report that many infections in married men, even in those with HIV-infected wives, could be acquired from outside the marriage [4]. Furthermore, a study conduct in South Africa to investigate who was infecting whom among migrant and non-migrant within concordance as well as discordance couples, found that non-migrant men were 10 times more likely to be infected from outside their regular relationships than inside [18].

Hence, it seems also relevant for Mozambican public health authorities to get more insights in this matter and to launch national prevention plans promoting regular HIV testing for couples (even prior to getting married or living together) and to support couples to prevent transmission once one partner has become infected. A similar recommendation was given by Kaiser *at al*. in Kenya, where they recommended that prevention interventions should begin early in relationships and include mutual knowledge of HIV status [5].

An interesting methodological topic for further research is the modification and application of the model to same-sex couples. Two approaches seem to be readily applicable, although further research is needed. A first possibility is the use of another unique identifier for both dimensions of the bivariate distribution. For instance, instead of *y*_1_ and *y*_2_ referring to the HIV status of woman and man respectively, one could use *y*_1_ to be the HIV status of the youngest of the two same-sex partners and *y*_2_ the status of the oldest of the two partners. Exactly the same model could be applied but now the two marginal success probabilities would represent the probabilities for the youngest partner and the oldest partner to be HIV positive. Any other unique identifier could be used, if available for all paired observations and if allowing interpretations of interest. A second approach would fully reflect the exchangeability. The HIV status of both partners could be put in any order to be *y*_1_ and *y*_2_. This would only make sense if both models for both probabilities would be taken exactly the same with all effects in common (not only partial as in our model), such that all data contribute to the estimation of that same probability *π* = *P*(*y*_1_ = 1|*x*) = *P*(*y*_2_ = 1|*x*). This would result in a model with only two components; the first two components of Eq (2) would be identical (*h*(*π*) = *β*^*T*^ *x*). As the SDM is symmetric, its definition Eq (6) can remain unchanged, but it reduces to (as both marginal probabilities are identical):

$$\text{SDM}=\frac{2(\pi -\pi_{11})}{2\pi -\pi_{11}}.$$

Similar modifications are to be applied to the joint probabilities Eq (3). What the potential as well as limitations of such an approach are, needs still to be investigated.

## Appendix: SAS code for the final CE-PE model (see results in Table 3)

proc nlmixed data = couples qpoints = 100;

/* Eq (8): probability model for P(y1 = 1) on logit scale (with random effect a) */

eta1 = b0_1 + b1_1*prev_d2 + b2_1*prev_d3 + b3_1*N_union_dW2 + b7_1*N_union_dM2 + b4_1*po + b5_1*mid + b8_1*Condom_dM1 + a;

/* probability model for p1_1 = P(y1 = 1) on probability scale */

p1_1 = 1 / (1 + exp(-eta1));

/* Eq (8): probability model for P(y2 = 1) on logit scale (with random effect a) */

eta2 = b0_1 + b1_1*prev_d2 + b2_1*prev_d3 + b3_2*N_union_dW2 + b7_2*N_Union_dM2 + b4_1*po + b5_1*mid + b8_2*Condom_dM1 + a;

/* probability model for p1_2 = P(y2 = 1) on probability scale */

p1_2 = 1 / (1 + exp(-eta2));

/* Eq (8): probability model for SDM on logit scale (without random effect) */

eta3 = b0_3 + b1_3*prev_d2 + b2_3*prev_d3 + b3_3*N_union_dW2;

SDM = 1/(1 + exp(-eta3));

/* probability model for SDM on probability scale */

/* Eqs (3)&(7) joint probabilities as function of p1_1 = P(y1 = 1), p1_2 = P(y2 = 1), SDM */

p11 = ((1-SDM)*(p1_1 + p1_2))/(2-SDM);

p10 = p1_1 − p11;

p01 = p1_2 − p11;

p00 = 1 − p1_1 − p1_2 + p11;

/* weighted log-likelihood for multinomial distribution (weights of survey design) */

ll = HIV_W*HIV_M*log(p11) + HIV_W*(1-HIV_M)*log(p10) + (1-HIV_W)*HIV_M*log(p01) + (1-HIV_W)*(1-HIV_M)*log(p00);

ll = ll*weight;

model ll ~ general(ll);

random a ~ normal(0,sigma2_a) subject = EA;

run;
